# Personal

**Published:** 1909-07

**Authors:** 


### PERSONAL.

Dr. Frederick Peterson, of New York, one of the oldest members of the National Conference of Charities and Correction, which recently held its annual meeting in Buffalo, left June 16, 1909, for a trip through the Orient. He is going to see how the Chinese and the Japanese take care of their insane and feeble-minded.

Dr. Thomas B. Osborne, of Louisville, a vice-president of the conference, will leave shortly for Europe. He will make a study of the charitable institutions of the continent.

Dr. William G. Taylor, of No. 576 Lafayette Avenue, Buffalo, was operated upon for appendicitis at the General Hospital recently. It is believed at this writing that his convalescence is assured.

Dr. John H. Grant, of Buffalo, has removed his residence from 380 Franklin Street, to 432 Delaware Avenue. His offices remain at the Mutual Life Building as heretofore.

Dr. Clayton M. Brown, of Buffalo, announces the removal of his office from 475 Franklin Street to 158 North Pearl, near North Street. Hours, 9 to 1. Bell telephone, Tupper 1037; Frontier, 3611.

Colonel William C. Gorgas, sanitary officer at Panama, president of the American Medical Association, received the honorary degree of doctor of laws at the recent annual commencement of Jefferson Medical College, Philadelphia.

Dr. Roswell Park, of Buffalo, delivered the principal address at the recent annual commencement of the Army Medical School at Washington.

Dr. Lewis S. McMurtry, of Louisville, was created doctor of laws at the May, 1909, commencement of Tulane University, New Orleans. Dr. McMurtry received his baccalaureate degree at Center College, Ky., in 1870, and graduated in medicine from Tulane University in 1873. A year later Center College conferred upon him the degree of master of arts. It must be pleasant thus to be honored twice by one's Alma Maters, and it certainly is most gratifying to the legion of friends of the recipient in this instance.

---

Dr. George F. Cott, of Buffalo, has announced the removal of his office from 1248 to 1195 Main Street, corner of Dodge. Dr. Chester C. Cott, also has established offices at the same location. Both telephones. Dr. Cott, Sr., was elected vice chairman of the section on laryngology and otology at the recent meeting of the American Medical Association, held at Atlantic City.

---

Dr. Walter A. Cowell, of Olean, was recently appointed health officer of that city.

---

Dr. William C. Krauss, of Buffalo, starts July 5, 1909, on a short trip to Europe, to return home about October first.

---

Dr. Joseph P. Brennan, an interne at the Buffalo General Hospital, has been appointed house surgeon at Bellevue, New York. Dr. Brennan secured his place through a competitive examination and will assume his new duties January 1, 1910.

